# Brain network dynamics in the wake-sleep transition reorganize according to task engagement

**DOI:** 10.1162/IMAG.a.1336

**Published:** 2026-09-08

**Authors:** Samika S. Kumar, Anat Arzi, Corinne A. Bareham, Javier Gonzalez-Castillo, Isabel Fernandez, Enzo Tagliazucchi, Pedro A.M. Mediano, Peter A. Bandettini, Tristan A. Bekinschtein

**Affiliations:** Consciousness and Cognition Lab, Department of Psychology, University of Cambridge, Cambridge, United Kingdom; Section on Functional Imaging Methods, Laboratory of Brain and Cognition, National Institute of Mental Health, Bethesda, MD, United States; Department of Medical Neurobiology and Department of Cognitive and Brain Science, The Hebrew University of Jerusalem, Jerusalem, Israel; Department of Clinical Neurosciences, University of Cambridge, Cambridge, United Kingdom; School of Psychology, Massey University, Auckland, New Zealand; Instituto de Física de Buenos Aires and Physics Department, University of Buenos Aires, Buenos Aires, Argentina; Department of Computing, Imperial College London, London, United Kingdom

**Keywords:** consciousness, cognition, alertness, vigilance, sleep onset

## Abstract

Alertness fluctuations and cognitive processing comprise overlapping brain circuits that include frontoparietal, thalamocortical, and sensory pathways. It is unclear how these brain circuits can simultaneously support such disparate but foundational human processes. To develop an integrated framework of consciousness and cognition, it is important to understand how fluctuations in alertness and cognitive processing interact in their shared circuits. In this electroencephalography (EEG)-functional magnetic resonance imaging (fMRI) study, we assessed individuals who fluctuated between alert and drowsy states while engaged in either an active or passive auditory task. We hypothesized that during periods of low alertness, individuals who actively maintain task engagement would recruit additional frontoparietal and sensory processing networks, while thalamocortical dynamics that typically change during sleep onset would remain unaffected. We found that when alertness decreased, passively listening to auditory tones led to increased synchronization primarily in the parietal lobe, whereas actively performing an auditory task resulted in increased long-range frontoparietal synchronization. During decreasing alertness, passive listening (but not active task engagement) was associated with widespread increased synchronization between the thalamus and cortex. In contrast, active task engagement (but not passive listening) led to increased synchronization between the auditory cortex and the rest of the brain. These results suggest the brain’s capacity for flexible functional reorganization when alertness levels decline but cognitive processes persist. The brain patterns that support transitions of consciousness at sleep onset also reflect ongoing cognitive task demands.

## 1 Introduction

Humans are often engaged in cognitive processes of varying attentional demand. In the background of these everyday attentional demands, alertness levels constantly fluctuate.

The same brain networks that give rise to cognitive processes are also recruited when alertness levels decline. Cortical networks, typically defined by the cognitive functions that they support, show dramatic changes at sleep onset, including widespread increased corticocortical functional connectivity (Spoormaker et al., 2010; Tagliazucchi & van Someren, 2017). Sleep onset also corresponds with increased functional connectivity in attentional networks but reduced long-range frontoparietal functional connectivity (Larson-Prior et al., 2009; Spoormaker et al., 2012). Frontoparietal networks, which are crucial for cognitive control and goal-oriented behavior (Marek & Dosenbach, 2018), also have dampened activity in other decreased states of consciousness (Laureys, 2005).

Sensory pathways are also engaged both during cognitive processing and during alertness fluctuations. Activation in the visual and auditory cortices, located in the occipital and temporal lobes, respectively, contributes to a brain-based prediction of alertness fluctuations (Falahpour et al., 2018; Goodale et al., 2021). At sleep onset, the capacity for auditory processing endures (Kouider et al., 2014), although it is accompanied with changes in auditory evoked potentials (Arzi et al., 2021; Winter et al., 1995). This suggests that when sensory processing is engaged at sleep onset, it may employ different mechanisms than those used during peak alertness levels.

Thalamocortical networks have been associated with cognitive processing and wake-sleep maintenance, respectively. The thalamus is considered an information processing hub that orchestrates bidirectional communication with different levels of the cortex (Gent et al., 2018; Sherman, 2016). This two-way thalamocortical dialogue appears to enable higher-level cognitive functions (Perry et al., 2023). In contrast, subjective reports of sleepiness coincide with less thalamocortical functional connectivity (Killgore et al., 2015). This is notably observed among sensory and motor areas, supporting the thalamus’s role in sensory gating during sleep (McCormick & Bal, 1994). At sleep onset, functional coordination between thalamus and cortex is severed, with the thalamus deactivating up to several minutes before the rest of the cortex (Magnin et al., 2010). The thalamus also heralds the return to wakefulness after sleep. When an individual awakens, the duration of subsequent thalamic activation predicts whether they will stay awake or drift back to sleep (Setzer et al., 2022).

Altogether, this evidence shows that frontoparietal, sensory, and thalamocortical pathways are separately critical for both cognitive processing and wake-sleep regulation. To develop an integrated understanding of how the capacity for cognition persists during fluctuating alertness, it is important to examine how these processes interact in their shared circuits (Goupil & Bekinschtein, 2012). Here, we used simultaneous EEG-fMRI to create an independence between the brain signal used to measure alertness level (i.e., electroencephalography [EEG]) and the brain signal used to test our hypotheses (i.e., functional magnetic resonance imaging [fMRI]). We focused our analysis on sleep onset: the consciousness state transition that spans high to low alertness levels (i.e., alert and drowsy states, respectively), and ends just before non-rapid eye movement (NREM) stage 2 sleep. We assessed frontoparietal, sensory, and thalamocortical networks because each has been frequently linked to consciousness, cognitive function, and alertness, but their contributions to overlapping aspects of these processes remain understudied. We hypothesized that when alertness levels decline towards a drowsy state: individuals who actively maintain task engagement recruit additional frontoparietal and sensory processing networks, while thalamocortical dynamics that typically change during sleep onset remain unaffected. We found that when alertness declined, passively listening to auditory tones led to increased synchronization mainly in the parietal lobe, whereas actively performing an auditory task resulted in increased long-range frontoparietal synchronization. During low alertness, passive listening (but not active task engagement) was associated with widespread increased synchronization between the thalamus and cortex. In contrast, active task engagement (but not passive listening) led to increased synchronization between the auditory cortex and the rest of the brain. We show here the brain’s capacity for flexible functional reorganization as alertness levels decline but cognitive processes persist. The brain patterns that support transitions of consciousness at sleep onset also reflect ongoing cognitive task demands.

## 2 Methods

This work was part of a larger preregistered project (https://osf.io/nua4y).

### 2.1 Data acquisition

**Fig. 1.**
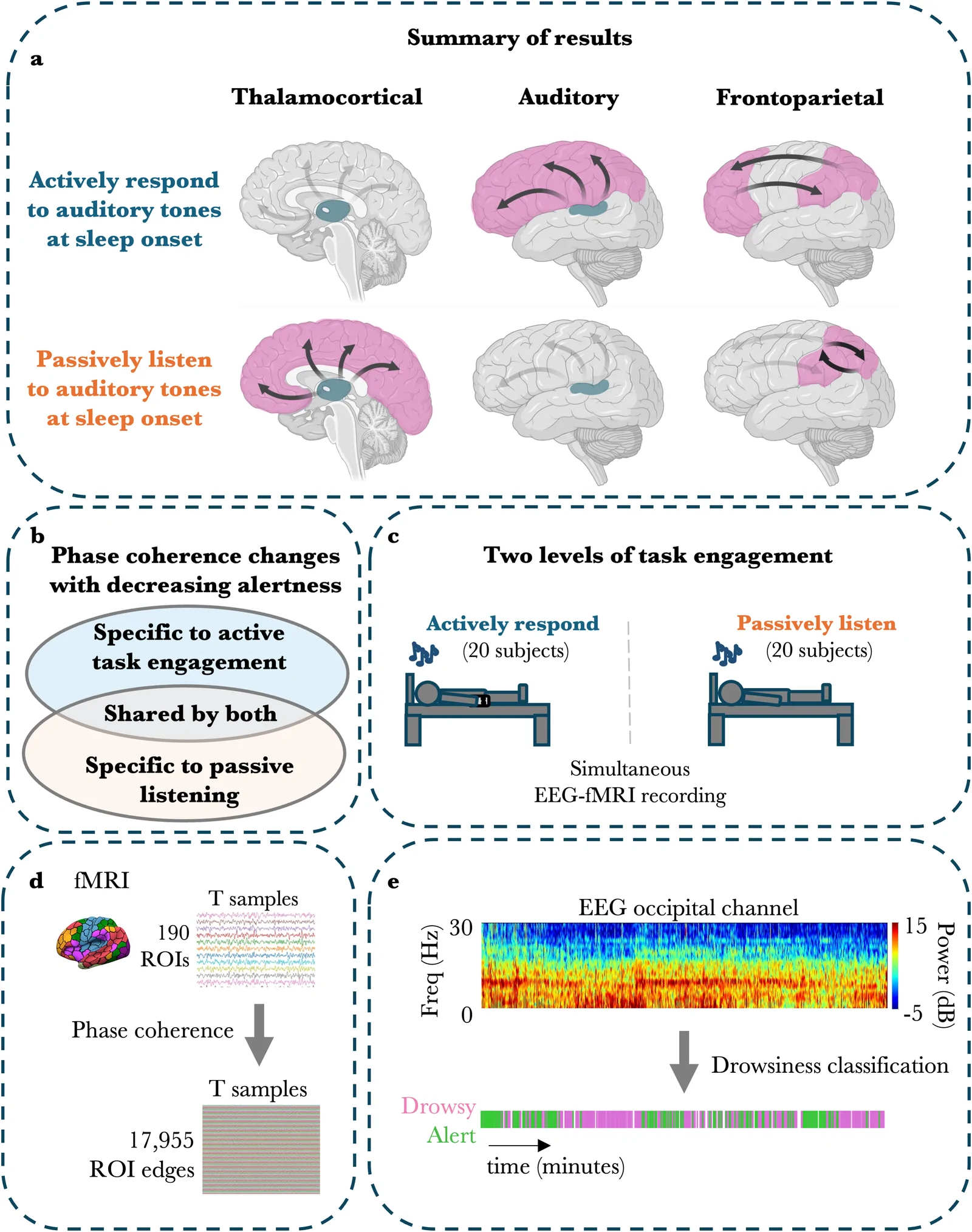
Experimental design, analysis, and summary of results. (a) Two separate groups of participants underwent EEG-fMRI recording while listening to auditory tones. One group actively responded to the tones while the other group passively listened. (b) EEG data epochs were classified as alert or drowsy. (c) The fMRI time series for 190 ROIs were extracted, and the instantaneous phase coherence was calculated between each pair of ROIs at each timepoint. (d) We explored changes in fMRI phase coherence that were specific to each level of task engagement and changes that were shared by both levels of task engagement. (e) Summary of main findings. At sleep onset, the *Active* task was associated with relatively unchanged thalamocortical dynamics, as well as increased auditory-cortical and frontoparietal synchronization. In contrast, the *Passive* task was associated with increased thalamocortical and parietal synchronization, and auditory-cortical dynamics were relatively unchanged.

This study comprised two datasets: (1) an *Active* task dataset in which participants actively responded to auditory tones using a button box, and (2) a *Passive* task dataset in which participants passively listened to auditory tones with no response requested (Fig. 1a). In both studies, EEG and fMRI were recorded concurrently. This way, alertness level and the co-occurring brain processes could be measured separately.

#### 2.1.1 Participants

In the *Active* task, 25 right-handed healthy volunteers (12 females; age 28 ± 4 years old) with normal hearing and no neurological or psychiatric conditions participated in the study. The *Passive* task had a different set of 26 volunteers (14 females; age 25 ± 5 years old) who were recruited with the same screening criteria. All participants were recruited using the Cambridge Psychology SONA system, provided informed consent prior to the study, and received a compensation of £10 per hour for participation. The protocols were approved by the Cambridge Psychological Research Ethics Committee before any testing began.

#### 2.1.2 Stimuli

Auditory tone stimuli were presented throughout the *Active* and *Passive* tasks.

*Active* task participants wore in-ear headphones, which presented harmonic complex tones that differed in their perceived location (ranging 0˚-60˚ left and right of midline). Tones were recorded in a free field prior to data collection using in-ear microphones. Participants were asked to indicate whether the tone originated from right or left of their midline by pushing a right- or left-side button on a response box with the right or left thumb, respectively.

*Passive* task participants passively listened to different sequences of auditory pure tones. Pure tone stimuli were presented in either ascending or descending order. Each tone was presented for 2 seconds. Once the sequence was completed, it restarted from either the highest or lowest frequency.

#### 2.1.3 Procedure

In preparation for the experiment, participants were instructed to refrain from drinking alcohol the night before and coffee on the day of the experiment. Before entering the MRI scanner, participants were fitted with an EGI electrolyte 128-channel cap developed by Electrical Geodesics, as well as earphones for stimulus presentation. In both tasks, participants were instructed to have their eyes closed.

In the *Active* task, participants were asked to respond as quickly and accurately as possible to the stimuli presented. In each stimulus trial, 5–8 seconds passed before the presentation of the tone, and the next trial began if a response was not detected within 5 seconds. Participants were encouraged to relax and not worry about falling asleep as the tone volume would increase and wake them up if needed. They were allowed about 5 minutes to relax and become sleepy, after which the session commenced. The entire task lasted approximately 50–60 minutes.

The *Passive* task comprised two sessions in randomized order that lasted an hour in total. In one session, participants were requested to remain awake, and in the other, they were encouraged to fall asleep. We did not account for these two different instructions in subsequent analyses because there was no difference between sessions according to participant self-report and post-hoc analyses of alertness level. Tones were presented during both sessions.

#### 2.1.4 Scanning acquisition parameters

Both tasks were conducted on the same Siemens 3T Prisma scanner using a 32-channel head coil for the *Active* task and a 20-channel head coil for the *Passive* task, both at the Medical Research Council Cognition and Brain Sciences Unit (MRC CBU) at the University of Cambridge. The 20-channel head coil is a larger coil, and it was selected to enable a wider range of participants to fit comfortably within the coil while wearing the EEG net. (This informed decision was made after data collection of the *Active* task.) EEG data were collected using the EGI Netstation software (www.egi.com) for both experiments.

In both tasks, T2*-weighted images were acquired with an echo-planar imaging (EPI) sequence using axial slice orientation (slice thickness: 3.75 mm; repetition time (TR): 2,000 ms; echo time: 30 ms; flip angle: 78˚; field of view: 192 × 192 mm; voxel size: 3 × 3 × 3.75 mm). A T1-weighted anatomical image was also acquired (TR: 2,250 ms; echo time: 2.98 ms; flip angle: 9˚; voxel size: 1 × 1 × 1 mm).

### 2.2 Data processing

#### 2.2.1 EEG preprocessing

EEG data acquired during fMRI scanning are subject to magnetic field gradient and ballistocardiographic (BCG) artifacts. We addressed these two artifacts using the EEGLAB plug-in provided by the University of Oxford Centre for Functional MRI of the Brain (FMRIB) (Delorme & Makeig, 2004; Niazy et al., 2005). In total, EEG preprocessing entailed the following steps. First, the gradient artifacts in the EEG data were identified using fMRI slice-timing information. An average artifact template was estimated from all gradient artifacts in the EEG signal, and this artifact template was subtracted from each gradient event. (The dynamic range of gradient artifacts was sufficient for this approach.) Residual gradient artifacts remained after this step, so a principal component analysis was then applied on all gradient events. The first four principal components were considered to reflect residual artifacts, and these were fitted and subtracted from each gradient event in the EEG signal. The resulting EEG data were downsampled to 250 Hz and filtered between 1–30 Hz. Manual artifact rejection was performed on the continuous EEG data to screen for non-BCG-related artifacts. To address BCG artifacts, an automatic QRS detection was performed on the electrocardiography (ECG) signal. These QRS events were aligned with the EEG data, and the corresponding BCG artifacts were removed with the same steps used to remove gradient artifacts. Finally, clean EEG data segments were entered in an independent component analysis to check for and remove noise-related artifacts that still remained. Missing and bad channels were interpolated using spherical spline interpolation.

#### 2.2.2 EEG-defined alertness

To measure alertness at an electrophysiological level, EEG data from both *Active* and *Passive* tasks were entered into a rule-based automatic algorithm for alertness classification, in which each 4-second data epoch was classified as “alert” or “drowsy” (i.e., high or low levels of alertness) (Jagannathan et al., 2018) (Fig. 1b). The algorithm is loosely based on the Hori system that breaks down the wake-sleep transition into nine stages (Tanaka et al., 1996). Briefly, the alert state corresponds with Hori stages 1 and 2, during which at least 50% of the EEG signal in an epoch has visibly high power in the alpha (8–12 Hz) frequency range. The drowsy state corresponds with Hori stages 3–8, during which the EEG signal flattens (Hori stages 3–4), gains power in lower frequency ranges (Hori stage 5), and develops vertex sharp waves and partial sleep spindles (Hori stages 6–8). NREM stage 2 sleep is reflected by Hori stage 9, during which complete sleep spindles emerge (Carskadon & Dement, 2011).

Here, the automatic algorithm developed by Jagannathan and colleagues assesses whole-brain information from EEG coherence and spectral power, as well as the presence of sleep grapho-elements, such as spindles and vertex sharp waves. This algorithm circumvents issues raised by the conventionally used EEG theta/alpha ratio, which is susceptible to contamination by frequencies reflecting cognitive processing. (Delta, theta, and alpha oscillations are also associated with high-level processes such as cognitive control, attention, and memory (Başar et al., 1999; Cavanagh & Frank, 2014; Harmony, 2013; Harmony et al., 1996; Klimesch, 1999; Putman et al., 2014).)

The algorithm has an accuracy (F1 score) of 85% for alert state classification and ~72% for drowsy state classification (Jagannathan et al., 2018), and it was originally designed to classify EEG data recorded outside the scanner. Here, after applying the algorithm on the cleaned EEG data recorded inside the scanner, we compared this classification with an EEG theta-alpha ratio computed on 4-second epochs from both datasets and the concurrent behavioral response data from the *Active* task dataset, as we expected the algorithm’s classification to reflect these conventional but coarser methods of alertness classification. Following this inspection, we adjusted weights on the automatic algorithm to better fit it for the simultaneous EEG-fMRI data.

The algorithm was trained and validated using 4-second epochs. We chose to use this same epoch length to mirror the preprocessing pipeline created by Jagannathan and colleagues and because alertness fluctuates on the order of seconds (Ogilvie, 2001).

#### 2.2.3 MRI preprocessing

MRI data were preprocessed using the Analysis of Functional NeuroImages toolbox (Cox, 1996) and FreeSurfer (http://surfer.nmr.mgh.harvard.edu/). We removed the first 10 volumes of each subject’s functional data. fMRI preprocessing steps included slice-time correction, motion correction, coregistration, normalization of the functional and structural data into standard space, spatial smoothing (4-mm full width at half maximum), and temporal filtering (0.01–0.1 Hz). (Because fMRI phase coherence is a less conventional measure than fMRI functional connectivity, we followed the same fMRI preprocessing pipeline used by Demertzi and colleagues (Demertzi et al., 2019), which included a bandpass filter. A bandpass filter has also been applied by others who have used fMRI phase coherence in their work (Mortaheb et al., 2022). Additionally, the instantaneous phase obtained from the Hilbert transform is only meaningfully interpretable when calculated on a narrowband signal because this allows the phase of the analytic signal to track a single underlying oscillatory component.)

A nuisance regression was performed using motion parameters, as well as the first three principal components from the ventricles and white matter (Behzadi et al., 2007; Jo et al., 2010). (Cerebrospinal fluid fluctuates with arousal (Gonzalez-Castillo et al., 2022), and these fluctuations can contaminate the fMRI gray matter signal and confound connectivity estimates.) We identified voxels that had low temporal variance (standard deviation < 3) to compute a group-level full-brain mask for subsequent analyses.

#### 2.2.4 fMRI analysis

The fMRI time series for regions of interest (ROIs) were extracted and visualized in Python v.3.8 (Python Software Foundation, http://python.org), and Nilearn (http://nilearn.github.io). We used a whole-brain cortical parcellation that divides the brain into seven networks and 200 parcels (Schaefer et al., 2018; Yeo et al., 2011). Voxel time courses were extracted for each parcel and spatially averaged to calculate the time series of a single ROI. We additionally extracted two time series for the left and right thalamus (Tzourio-Mazoyer et al., 2002). The group-level mask had poor coverage in the frontal and temporal poles, coinciding with the Schaefer/Yeo Limbic Network. These 12 Limbic ROIs were consequently removed from analyses, so there were 188 cortical + 2 thalami = 190 ROIs total. We were interested in functional changes between the auditory cortex and the rest of the brain, so we extracted four ROIs from the Schaefer/Yeo Somatomotor Network to create an Auditory module (*17Networks_LH_SomMotB_Aud1 / Aud2, 17Networks_RH_SomMotB_Aud1 / Aud2*).

Our method to calculate fMRI phase coherence has been previously reported (Demertzi et al., 2019). Briefly, we first computed the analytic signal of each ROI time series by applying a Hilbert transform. This analytic signal can be decomposed into an instantaneous phase and amplitude at each time point. (The instantaneous phase results from the contribution of all frequencies embedded in the signal at a given instant.) We obtained the instantaneous phase (between -π and +π) of each ROI time series at each timepoint, and we calculated the cosine of the difference in phases between each pair of ROI time series. The result was a 190 × 190 phase coherence matrix at each timepoint that contained a phase coherence value for every pair of ROIs (Fig. 1c). Phase coherence values ranged between -1 and +1.

To maximize comparability between *Active* and *Passive* task analyses, we did not model auditory stimuli or responses in the fMRI analysis. A stimulus-independent, model-free analysis allowed us to capture spontaneous intrinsic brain patterns that reflect the distinct cognitive and internal states unique to each task condition.

#### 2.2.5 Data selection criteria

We applied several data selection criteria to ensure that data from the two tasks were comparable. In the *Active* task, participants’ continuous behavioral responses confirmed that they had not entered NREM stage 2 sleep, which is characterized by unresponsiveness (Carskadon & Dement, 2011). In the *Passive* task, participants were susceptible to falling into NREM light sleep. For the statistical analyses, we selected *Passive* task runs that only comprised wake and/or NREM1 according to manual sleep staging.

Since fMRI phase coherence captures signal dynamics at each timepoint, it is imperative that each data point entered into the analysis is not contaminated by motion artifacts. In addition to motion correction in our preprocessing pipeline, we applied a strict motion threshold for admitting data points into the analysis. With the fMRI data, a Euclidean norm was calculated from the six motion parameters to get a single metric of motion at each timepoint. We excluded each high-motion volume (>.25 mm), as well as one data point before and after (12.1 ± 2.4% volumes removed per *Active* task subject; 5.7 ± 1.9% volumes removed per *Passive* task subject). The *Active* task subjects had significantly more volumes removed due to high-motion criteria than *Passive* task subjects (*t* = 2.09, *p* = 0.04). However, we found no significant difference in the Euclidean norm of the six motion parameters after applying our high-motion exclusion criteria (*t* = 1.61, *p* = 0.11; *Active* Euclidean norm: 0.07 ± 0.00 mm; *Passive* Euclidean norm: 0.06 ± 0.00 mm).

Given that the exact EEG-fMRI temporal lag can vary across different parts of the brain, we performed two steps to try to ensure that a given fMRI data point transpired during an EEG-defined alert or drowsy state. First, we delayed the fMRI time series by 3 timepoints (6 seconds) because it has been estimated that the BOLD signal follows neural processes within the range of a 4- to 6-second delay (Liao et al., 2002; Magri et al., 2012). Second, we selected fMRI data points that remained in the same EEG alertness state for both the current and subsequent timepoint. This meant that each fMRI data point that was selected for the primary analysis would have transpired during the same EEG alertness state regardless of how much the true EEG-fMRI temporal lag varied within a 4- to 6-second timeframe.

This final subset of the data was used for all statistical analyses. Following these data selection criteria, the *Active* task had 7,031 total samples assigned as alert (351.55 ± 126.35 samples per subject) and 6,931 total samples assigned as drowsy (346.55 ± 174.79 samples per subject), which were entered into the statistical analysis. The *Passive* task had 8,568 total samples assigned as alert (428.40 ± 213.38 samples per subject) and 10,433 total samples assigned as drowsy (521.65 ± 244.93 samples per subject), which were entered into the statistical analysis. After applying data selection criteria, consecutive same-state segments in the *Active* task had a mean length of 3.22 ± 4.54 samples in the alert state and a mean length of 3.21 ± 3.97 samples in the drowsy state. Consecutive same-state segments in the *Passive* task had a mean length of 3.55 ± 4.74 samples in the alert state and a mean length of 3.97 ± 5.26 samples in the drowsy state (Supplementary Fig. S1).

Following the aforementioned strict criteria for data selection, the data points were not necessarily temporally adjacent within-subject, so we could not model temporal dependence between timepoints. Instead, within-subject data points were treated as independent and isolated in the statistical analyses.

#### 2.2.6 Statistical analysis

We identified alertness state-dependent changes in fMRI phase coherence that were common to both *Active* and *Passive* tasks, as well as those that were specific to each task (Fig. 1d). We used Bayesian statistical evidence to identify these functional changes. For each unique pair of ROIs (i.e., each ROI edge), we quantified the effect of State (drowsy versus alert) predicting phase coherence in a linear mixed-effects model, with subject as random effect: *phase_coherence ~ State + (1|subject)* (Supplementary Fig. S2a). Our null model only included subject as random effect (i.e., *phase_coherence ~ (1|subject)*). We used the natural log (*ln*) of the Bayes Factor (BF) to conservatively constrain the results to ROI edges that had sufficient evidence in favor of the *State* model’s prediction (i.e., *ln(BF_State model_) > 2*, roughly equivalent to *BF_State model_ > 7*, when *ln(BF_null_) = 0*) (Andraszewicz et al., 2015; Jeffreys, 1961; Kass & Raftery, 1995) (Supplementary Fig. S2b). (A BF is used to compare two statistical models against each other, and it quantifies the likelihood that one model (i.e., *State* model) describes the data better than the other model (i.e., null model). A higher BF indicates greater support in favor of the *State* model (Morey et al., 2016).)

As there is no agreed-upon method to correct for multiple tests in Bayesian statistics, we chose to employ a more conservative (i.e., higher) Bayes Factor threshold so that greater evidence was required to reject the null model. We selected a Bayes Factor threshold (i.e., 7) that demarcates the upper end of the Bayes Factor category for moderate, or substantial, evidence (i.e., Bayes Factor between 3 and 10) (Jeffreys, 1961; Lee & Wagenmakers, 2014). Others have used the upper end of this Bayes Factor category as the threshold for sufficient statistical evidence as well (Pascovich et al., 2022). For each ROI edge, we identified whether either one or both tasks had sufficient evidence in favor of the *State* model (Supplementary Fig. S3). If both tasks had sufficient evidence in favor of the State model for a given ROI edge and statistical contrast, then this ROI edge was considered “shared” by both levels of task engagement.

Statistical analyses were performed in R v.4.0, using the libraries lmerTest (Kuznetsova et al., 2017) and bayestestR (Makowski et al., 2019).

The diagram summary (Fig. 1e) was created with BioRender.com, and glass brain results were created with Nilearn (http://nilearn.github.io). Analyses were conducted using computational resources from the NIH HPC Biowulf cluster (http://hpc.nih.gov).

## 3 Results

We characterized how auditory, thalamocortical, and frontoparietal networks changed when alertness decreased during two levels of auditory task engagement (Fig. 1e). In the following comparisons, we identified ROI edges that had increased phase coherence in the *Drowsy > Alert* contrast (Supplementary Table S1).

### 3.1 Low alertness shows increased thalamocortical synchronization during passive listening but not during active task engagement

**Fig. 2.**
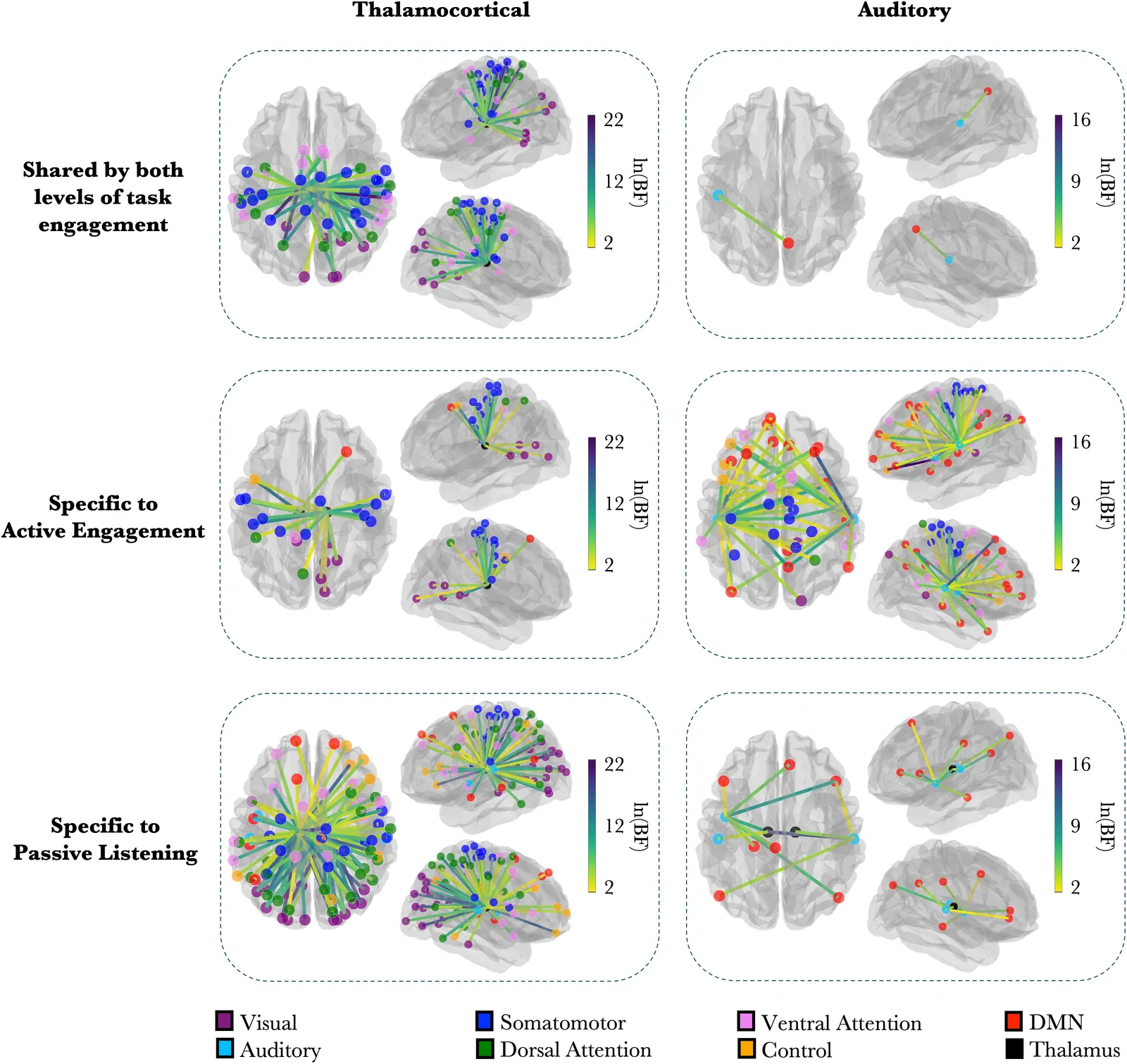
Spatial topography of thalamocortical and auditory-cortical phase coherence. (Thalamocortical column) Glass brains show the thalamocortical network edges that had sufficient evidence in favor of the *State* model for the *Drowsy > Alert* contrast (see Methods). Edge color denotes the BF. Node color denotes the ROI’s network. **(Auditory column)** Glass brains show the network edges with the Auditory module that had sufficient evidence in favor of the *State* model for the *Drowsy > Alert* contrast. **(Top row)** Changes in phase coherence that were shared by both levels of task engagement. **(Middle row)** Phase coherence changes specific to the *Active* task. **(Bottom row)** Phase coherence changes specific to the *Passive* task.

We examined how thalamocortical networks change when alertness declines because they typically mark the passive descent from wakefulness to sleep (Hill & Tononi, 2005) but also support cognitive functions (Perry et al., 2023). With decreasing alertness, passive listening was associated with stronger and more widespread thalamocortical synchronization, in comparison to active task engagement. For both tasks, we observed increased phase coherence in the *Drowsy > Alert* contrast between bilateral thalami and specific cortical (i.e., Visual, Somatomotor, Dorsal Attention, Ventral Attention) networks (for [number of edges that passed threshold / total number of available edges] 61/216 edges; Active ln(BF): 7.34 ± 0.62; Passive ln(BF): 10.23 ± 0.76) (Fig. 2: Thalamocortical, top row). These thalamocortical network edges included nodes distributed around the precentral and postcentral gyri (Somatomotor Network), supramarginal gyrus and posterior part of the cingulate gyrus (Ventral Attention Network), superior parietal lobule (Dorsal Attention Network), and occipital regions of the Visual Network.

However, we also found additional widespread increased thalamocortical phase coherence that was specific to the *Passive* task in the *Drowsy > Alert* contrast across all cortical networks (for 107/376 edges; Passive ln(BF): 7.86 ± 0.43) (Fig. 2: Thalamocortical, bottom row), although with higher distribution in parietal and occipital regions. Thalamocortical increases in phase coherence that were shared by both tasks, as well as those specific to the *Passive* task, largely stemmed from the left thalamus (Supplementary Fig. S4, Thalamocortical).

We found few thalamocortical network edges that had increased phase coherence during the *Active*, but not *Passive*, task in the *Drowsy > Alert* contrast (for 26/368 edges; Active ln(BF): 6.53 ± 0.69) (Fig. 2, Thalamocortical, middle row). These edges extended to the precentral and postcentral gyri of the Somatomotor Network, as well as the lingual gyrus of the Visual Network. They largely stemmed from the right thalamus (Supplementary Fig. S4, Thalamocortical).

### 3.2 Low alertness shows increased auditory-cortical synchronization during active task engagement but not during passive listening

We anticipated changes in phase coherence between the auditory cortex and the rest of the brain during declining alertness because both tasks in this study involved auditory stimuli, and auditory-related functional reorganization has been inferred between wakefulness and sleep stages (Arzi et al., 2021). We found that with declining alertness, active task engagement, but not passive listening, was associated with widespread auditory-cortical synchronization. This increased phase coherence in the *Drowsy > Alert* contrast during the *Active*, but not *Passive*, task spanned all cortical networks (for 56/736 edges; Active ln(BF): 4.78 ± 0.37) (Fig. 2: Auditory, middle row). The auditory-cortical edges with the strongest increase in phase coherence were largely distributed around the bilateral supplementary motor cortex (Somatomotor Network); left frontal pole (Control Network); right superior frontal gyrus and right precuneus (Default Mode Network (DMN)); and right insula (Ventral Attention Network). This increased phase coherence with left and right auditory nodes was equally distributed across the ipsilateral and contralateral hemispheres (Supplementary Fig. S4, Auditory).

In comparison, we found minimal auditory-cortical synchronization during decreasing alertness that was specific to passive listening. These auditory-cortical edges that had increased phase coherence in the *Drowsy > Alert* contrast during the *Passive*, but not *Active* task, were distributed in the DMN and bilateral thalami (for 12/192 edges; Passive ln(BF): 5.36 ± 0.90) (Fig. 2, Auditory, bottom row).

There was only one auditory-cortical edge shared by both *Active* and *Passive* tasks that had increased phase coherence in the *Drowsy > Alert* contrast (1/184 edges; Active ln(BF): 2.573; Passive ln(BF): 5.892) (Fig. 2, Auditory, top row).

### 3.3 Low alertness shows frontoparietal synchronization during active task engagement but predominantly parietal synchronization during passive listening

**Fig. 3.**
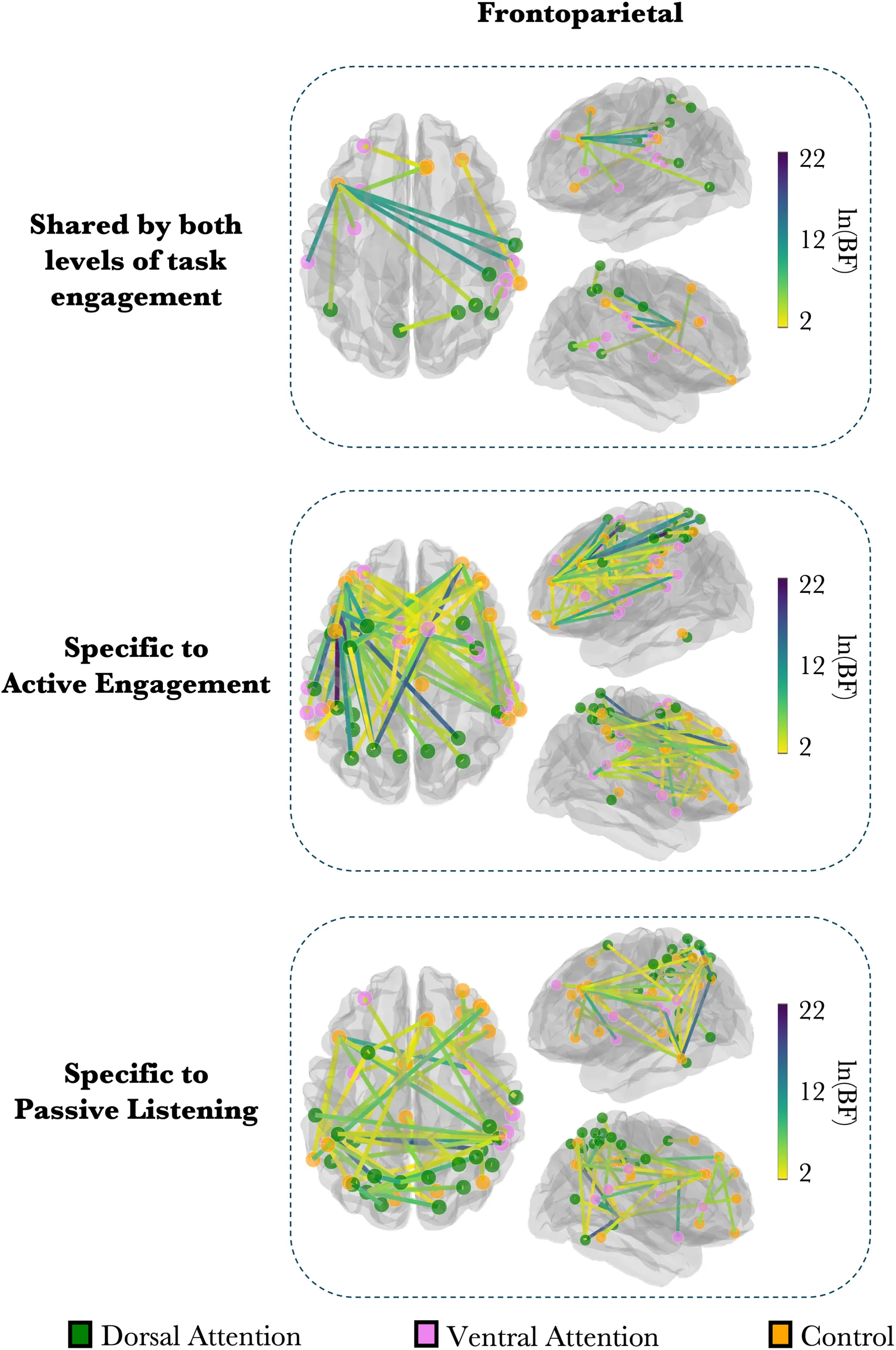
Spatial topography of frontoparietal network phase coherence. Glass brains show the frontoparietal network edges (within the Dorsal Attention, Ventral Attention, and Control Networks) that had sufficient evidence in favor of the *State* model for the *Drowsy > Alert* contrast. Edge color denotes the BF. Node color denotes the ROI’s network. **(Top row)** Changes in phase coherence that were shared by both levels of task engagement. **(Middle row)** Phase coherence changes specific to the *Active* task. **(Bottom row)** Phase coherence changes specific to the *Passive* task.

Finally, we explored the spatial topography of frontoparietal synchronization during decreasing alertness because we expected increased frontoparietal synchronization (i.e., among Dorsal Attention, Ventral Attention, and Control Networks) to reflect the interaction between decreasing alertness and sustained decision-making (Canales-Johnson et al., 2020; Czisch et al., 2012; Jagannathan et al., 2022). We found that active task engagement, but not passive listening, was associated with long-range frontoparietal synchronization during decreasing alertness. These long-range frontoparietal network edges that had increased phase coherence in the *Drowsy > Alert* contrast during the *Active*, but not *Passive*, task, extended from medial, frontopolar, and dorsolateral prefrontal areas toward bilateral posterior parietal hubs (82 edges; ln(BF): 5.565 ± 0.448) (Fig. 3, middle row).

In comparison, decreasing alertness during passive listening was associated with more parietal synchronization. We found fewer frontoparietal network edges and more posterior parietal edges that had increased phase coherence in the *Drowsy > Alert* contrast during the *Passive*, but not *Active*, task. This increased phase coherence included a heavily connected interhemispheric network of posterior parietal and temporoparietal edges and little frontal involvement (48 edges; ln(BF): 5.491 ± 0.49) (Fig. 3, bottom row).

We found that with decreasing alertness, little frontoparietal synchronization was common to both levels of task engagement. That is, *Active* and *Passive* tasks only shared a few common frontoparietal network edges that had increased phase coherence in the *Drowsy > Alert* contrast (13 edges; Active ln(BF): 7.544 ± 1.625; Passive ln(BF): 5.025 ± 0.568) (Fig. 3, top row).

## 4 Discussion

Here, we showed how thalamocortical, auditory, and frontoparietal dynamics change during declining alertness across two levels of task engagement: one that engaged only sensory processing (*Passive* task) and a second that additionally required perceptual decision-making, evidence accumulation and prediction, as well as action initiation (*Active* task). We found stronger long-range frontoparietal and auditory-cortical synchronization with decreasing alertness when participants were actively engaged in an auditory task, but not when passively listening to auditory tones. With decreasing alertness, passive listening, but not active task engagement, was associated with increased synchronization within the parietal lobes and widespread increased thalamocortical synchronization. Alertness fluctuations and cognitive processing are associated with overlapping brain dynamics. How these two processes jointly modulate their overlapping brain circuits is needed to advance towards an integrated understanding of consciousness and cognition (Goupil & Bekinschtein, 2012).

We found widespread changes in thalamocortical networks during the *Passive* task when alertness declined. However, the *Active* task only had a partial thalamocortical functional reorganization. **Thalamocortical** networks have been associated with the early process of falling asleep (Magnin et al., 2010) and losing consciousness during sedation (Barttfeld et al., 2015), with the thalamus leading the cortex in this transition (Baker et al., 2014). However, it has also been shown that a functional thalamus is not necessary for general wake-sleep maintenance (Hindman et al., 2018). It appears that thalamocortical dynamics correspond with processes that transpire during either wakefulness or sleep (McCormick & Bal, 1994; Setzer et al., 2022), but might not directly cause wakefulness or sleep (Gent et al., 2018). Our work furthers this premise: we show that different levels of cognitive processing during sleep onset coincide with different profiles of thalamocortical synchronization. We speculate that the largely unchanged thalamocortical dynamics during active task engagement indicate active cognitive processes prevailing into the drowsy state. This is supported by EEG studies, which show that brain networks that are active during a cognitive task remain active even if the participant is fluctuating in alertness (Comsa et al., 2019; Jagannathan et al., 2022) or in light sleep (Kouider et al., 2014). A recent study conveyed this same idea in another way by showing that thalamocortical dynamics are preserved when alertness fluctuations are accounted for during fMRI task performance (Goodale et al., 2021).

This work was part of a preregistered project (https://osf.io/nua4y), where we initially hypothesized that the *Active* task would show higher thalamocortical synchronization in the *Drowsy > Alert* contrast in comparison to the *Passive* task. This notion was based on early literature that reported thalamocortical decoupling during sleep onset (Magnin et al., 2010). We expected that the alert-drowsy transition in the *Passive* task would more closely resemble uninterrupted sleep onset than the *Active* task, so it would therefore have more thalamocortical decoupling. After the publication of this preregistration, several recent studies led us to revise our hypotheses. First, a few papers described thalamocortical networks’ role in maintaining cognitive processes (Hwang et al., 2022; Shine et al., 2023), which led us to hypothesize that participants in the *Active* task would actually have thalamocortical networks that stay active during both alert and drowsy states. Second, other papers described corticothalamocortical networks that orchestrate the brain’s global shifts in cortical synchrony (Portoles et al., 2022; Soplata et al., 2023), suggesting that the widespread cortical synchronization at sleep onset (Spoormaker et al., 2010; Tagliazucchi & van Someren, 2017) may additionally coincide with thalamocortical synchronization. Hence, we revised our hypotheses to predict that with declining alertness, the *Passive* task would have increased thalamocortical synchronization in comparison to the *Active* task.

It has been long proposed that large-scale **frontoparietal organization** underlies the integration of cognitive processes from cross-network interaction (Duncan, 2010; Marek & Dosenbach, 2018; Naghavi & Nyberg, 2005; Stokes et al., 2013). We recently proposed that this integration separately depends on both alertness level and task (Mediano et al., 2021). Here, we show the brain’s flexibility to functionally reorganize during decreasing alertness according to cognitive task load. We interpret our findings with a cognitive framework of neural information integration (Toker & Sommer, 2019), whereby phase coherence reflects the brain network resources required to sufficiently integrate information. The increased frontoparietal synchronization observed in the *Drowsy > Alert* contrast during the *Active*, but not *Passive*, task suggests that during low alertness, additional cognitive resources are required to achieve the level of information integration that is associated with high alertness. These frontoparietal network edges did not show increased phase coherence in the *Drowsy > Alert* contrast during the *Passive* task, suggesting that they contribute specifically to active task engagement in the drowsy state and are not driven by sleep onset alone. This notion is backed by findings that link slower reaction time response with higher frontoparietal activation (Bogler et al., 2017; Jagannathan et al., 2022).

In contrast, the predominantly parietal lobe synchronization in the *Drowsy > Alert* contrast during the *Passive* task indicates that minimal brain resources are allocated in response to hearing auditory tones played at sleep onset when attention is not sustained. Our results support the notion that the brain operates flexibly and has available resources to recruit when challenged (Price & Friston, 2002). This notion is in line with the cognitive reserve hypothesis, which also posits that the brain has flexibly available “reserves” to compensate for other dysfunction (Colangeli et al., 2016). Here, this compensation is indicated by frontoparietal networks that are available to be differentially deployed according to task engagement. We bridge the cognitive reserve hypothesis with the alert-drowsy transition to suggest that the brain’s capacity to maintain cognition during alertness fluctuations is driven by flexibly allocated, compensatory reserves in a frontoparietal system.

**Auditory** processing is largely preserved in sleep (Blume et al., 2018; Kouider et al., 2014), but we have recently suggested that a functional reorganization occurs across wakefulness and sleep stages (Arzi et al., 2021). Our work mechanistically confirms that such reorganization takes place, and we show that it begins as early as the transition from high to low alertness and that it depends on task engagement. When alertness decreased, auditory-cortical synchronization increased most during the *Active* task, particularly among prefrontal brain areas and the Somatomotor Network. The prefrontal cortex underpins high-level cognitive processes such as decision-making (Krawczyk, 2002), while the Somatomotor Network supports motor tasks (Desmurget & Sirigu, 2009; van den Heuvel & Hulshoff Pol, 2010). In the *Active* task, participants were not only hearing tones, but also making a decision to press one of two buttons, thus maintaining perceptual decision-making processes (Xu et al., 2023). We conclude that the mechanisms for motor task engagement and auditory integration interconnected more when alertness faded.

The relevance of our results is backed by the pervasiveness of alertness fluctuations in daily life. Throughout the day, alertness fluctuations depend on a multiplicity of factors (Meyer et al., 2022). Tedious tasks and passive engagement can drive mind-wandering and mind-blanking, both of which are linked with low alertness levels (Andrillon et al., 2021; Bonsignore et al., 2022; Jubera-Garcia et al., 2021). These brain states correspond with increased synchronization across brain regions (Mortaheb et al., 2022), which is supported by our results (Supplementary Fig. S3).

In the current study, we took advantage of the natural tendency of our participants to frequently fluctuate between high and low alertness due to either a boring, tedious task (i.e., *Active* task) or a lack of engagement (i.e., *Passive* task). We labeled their states as alert or drowsy based on robust computational methods applied to the EEG signal dynamics (Jagannathan et al., 2018). While others have successfully used either simultaneous EEG-fMRI to detect arousal-related behavioral changes (Goodale et al., 2021) or fMRI alone to characterize states during the passive transition to sleep (Tagliazucchi & Laufs, 2014), we decided here to create an independence between the brain signal that was used to measure alertness (i.e., EEG) and the brain signal that was used to test our hypotheses (i.e., fMRI). We used fMRI phase coherence to characterize the brain as a complex system with nonlinear interactions between wakefulness and cognitive task engagement (Song & Tagliazucchi, 2020). There is an established view of the dynamics across different levels of task engagement, along with the anticorrelations observed among large brain networks (Fox et al., 2005; Hutchison et al., 2013), which needs to be integrated with the literature on wakefulness versus sleep for subcortical and cortical networks (Tagliazucchi & van Someren, 2017). In this study, we offer the same cognitive neuroscience framework for both, conceptually and analytically. While apparently overlapping frontoparietal networks have been systematically implicated in executive function, decision-making, and cognitive control tasks (Duncan, 2013), the same networks have been signaled as key for decreased consciousness states such as sleep, anesthesia, vegetative state, and minimally conscious state (Laureys, 2005). This apparent field split between cognition and consciousness (Martin et al., 2021) is directly put to the test in the current experiments, showing a nuanced interaction between task engagement and transitions to lower consciousness states. We found moderate evidence that active and passive task engagement yield different changes in phase synchronization between alert and drowsy states (Figs. 2 and 3), but the magnitude of these differences is small (Supplementary Table S1). Therefore, these differences may have little clinical relevance, but they are noteworthy for scientific understanding.

### 4.1 Limitations and future work

Cautious interpretation should be made with indirect neural measures. This work relied on fMRI phase coherence as a measure of dynamic brain regional functional coordination. We favored phase coherence over functional connectivity as it is an instantaneous measure, and alertness fluctuations vary on the timescale of seconds (Jagannathan et al., 2018). Phase coherence has also been used successfully in other recent work (Chang & Glover, 2010; Demertzi et al., 2019; Glerean et al., 2012; Mortaheb et al., 2022; Zarghami et al., 2020). However, follow-up work could confirm the stability of our findings on longer timescales.

Follow-up data collection could also include other physiological measures (e.g., photoplethysmography, respiration), which could be used to account for physiological processes that naturally fluctuate with alertness. Here, in our fMRI preprocessing pipeline, we applied the CompCor method (Behzadi et al., 2007), a technique that is commonly used to account for physiological noise when respiratory traces are not available.

#### 4.1.1 Active and Passive task differences

In addition to the level of task engagement, the *Active* and *Passive* tasks had some other differences. This study was performed with two independent groups of participants, meaning that differences in task effects could be influenced by differences between the participant groups. Furthermore, the stimuli presented in our two tasks had several different low-level properties (e.g., tone frequency, tone sequence and presentation rate, presence of a spatial component). Follow-up work should involve a within-subject experimental design in which the same set of participants perform different tasks during alertness fluctuations. Additionally, only one property of the task stimulus would ideally be manipulated in each task condition to ascertain that the brain profile truly reflects the interaction between task condition and alertness level.

Also, because the *Active* and *Passive* task datasets were collected with different head coils, coil-related variation in signal sensitivity across brain regions may have contributed to some between-dataset differences. While our ROI-based approach possibly reduced some susceptibility to this issue, ideally such datasets would be acquired under identical hardware conditions in future work.

Here, we addressed having several differences between the tasks through our analyses. Instead of analyzing a true statistical interaction between Dataset and State, we first compared alert and drowsy states within each dataset, as we anticipated that some attributes unique to each dataset would be canceled out.

Furthermore, the *Active* and *Passive* task datasets had differences in their experimental designs that we believe allowed us to isolate the process of sustained attention. In the *Active* task, tones were presented with a long and varying inter-stimulus interval (ISI), meaning that the task involved sustained attention (Seli et al., 2012). The *Active* task also had a spatial component in that tones were presented from varying spatial directions. In comparison, the *Passive* task consisted of a sequence of pure tones that were played continuously in an expected order. We speculate that when an auditory stimulus has an unpredictable spatial component, as well as an unpredictable ISI, it pulls individuals’ attention due to the continuous novelty of a changing stimulus source. A more repetitive stimulus with a constant spatial source for the *Passive* task may have aided true passive task engagement.

#### 4.1.2 Incorporating behavior and more states of consciousness

The brain network dynamics identified in active task engagement during low alertness may not be sufficient to maintain cognition in the same way that it is maintained during high alertness (Canales-Johnson et al., 2020; Jagannathan et al., 2022). While some brain functional dynamics may allow us to perform at a nearly optimal level when drowsy, others may be maladaptive and correspond with poor performance and pathological mechanisms. Therefore, while our results may suggest a causal linkage between behavior and different brain network dynamics modulated by alertness, this causality requires further exploration (Krakauer et al., 2017).

This work examined auditory task engagement during the alert-drowsy transition. However, the brain continuously engages in functional reorganization during all transitions in consciousness (e.g., from wakefulness into sedation (Barttfeld et al., 2015), in disorders of consciousness (Demertzi et al., 2019), and upon entering a psychedelic-induced state (Mortaheb et al., 2024)). A clear next step would be to study different levels of task engagement across a broader range of consciousness transitions in order to fully characterize how conscious level and cognitive function interact.

## Supplementary Material

Supplementary Material

## Data Code and Availability

The analysis script and anonymized data are available at https://osf.io/nua4y.

## References

[IMAG.a.1336-b1] Andraszewicz, S., Scheibehenne, B., Rieskamp, J., Grasman, R., Verhagen, J., & Wagenmakers, E.-J. (2015). An introduction to Bayesian hypothesis testing for management research. Journal of Management, 41(2), 521–543. 10.1177/0149206314560412

[IMAG.a.1336-b2] Andrillon, T., Burns, A., Mackay, T., Windt, J., & Tsuchiya, N. (2021). Predicting lapses of attention with sleep-like slow waves. Nature Communications, 12(1), 3657. 10.1038/s41467-021-23890-7 PMC8241869 34188023

[IMAG.a.1336-b3] Arzi, A., Trentin, C., Laudini, A., Krugliak, A., Nikolla, D., & Bekinschtein, T. (2021). Dynamic auditory remapping across the sleep-wake cycle. bioRxiv. 10.1101/2021.02.16.431383

[IMAG.a.1336-b4] Baker, R., Gent, T. C., Yang, Q., Parker, S., Vyssotski, A. L., Wisden, W., Brickley, S. G., & Franks, N. P. (2014). Altered activity in the central medial thalamus precedes changes in the neocortex during transitions into both sleep and propofol anesthesia. Journal of Neuroscience, 34(40), 13326–13335. 10.1523/jneurosci.1519-14.2014 25274812 PMC4180471

[IMAG.a.1336-b5] Barttfeld, P., Bekinschtein, T. A., Salles, A., Stamatakis, E. A., Adapa, R., Menon, D. K., & Sigman, M. (2015). Factoring the brain signatures of anesthesia concentration and level of arousal across individuals. NeuroImage: Clinical, 9, 385–391. 10.1016/j.nicl.2015.08.013 26509121 PMC4588413

[IMAG.a.1336-b6] Başar, E., Başar-Eroğlu, C., Karakaş, S., & Schürmann, M. (1999). Are cognitive processes manifested in event-related gamma, alpha, theta and delta oscillations in the EEG? Neuroscience Letters, 259(3), 165–168. 10.1016/s0304-3940(98)00934-3 10025584

[IMAG.a.1336-b7] Behzadi, Y., Restom, K., Liau, J., & Liu, T. T. (2007). A component based noise correction method (CompCor) for BOLD and perfusion based fMRI. NeuroImage, 37(1), 90–101. 10.1016/j.neuroimage.2007.04.042 17560126 PMC2214855

[IMAG.a.1336-b8] Blume, C., Del Giudice, R., Wislowska, M., Heib, D. P. J., & Schabus, M. (2018). Standing sentinel during human sleep: Continued evaluation of environmental stimuli in the absence of consciousness. NeuroImage, 178, 638–648. 10.1016/j.neuroimage.2018.05.056 29859261

[IMAG.a.1336-b9] Bogler, C., Vowinkel, A., Zhutovsky, P., & Haynes, J.-D. (2017). Default network activity is associated with better performance in a vigilance task. Frontiers in Human Neuroscience, 11, 623. 10.3389/fnhum.2017.00623 29311878 PMC5743927

[IMAG.a.1336-b10] Bonsignore, M. R., Lombardi, C., Lombardo, S., & Fanfulla, F. (2022). Epidemiology, physiology and clinical approach to sleepiness at the wheel in OSA patients: A narrative review. Journal of Clinical Medicine Research, 11(13), 3691. 10.3390/jcm11133691 PMC9267880 35806976

[IMAG.a.1336-b11] Canales-Johnson, A., Beerendonk, L., Blain, S., Kitaoka, S., Ezquerro-Nassar, A., Nuiten, S., Fahrenfort, J., van Gaal, S., & Bekinschtein, T. A. (2020). Decreased alertness reconfigures cognitive control networks. Journal of Neuroscience, 40(37), 7142–7154. 10.1523/jneurosci.0343-20.2020 32801150 PMC7480250

[IMAG.a.1336-b12] Carskadon, M. A., & Dement, W. C. (2011). Chapter 2 – Normal human sleep: An overview. In M. H. Kryger, T. Roth, & W. C. Dement (Eds.), Principles and practice of sleep medicine (pp. 16–26). Elsevier Saunders. 10.1016/b978-1-4160-6645-3.00002-5

[IMAG.a.1336-b13] Cavanagh, J. F., & Frank, M. J. (2014). Frontal theta as a mechanism for cognitive control. Trends in Cognitive Sciences, 18(8), 414–421. 10.1016/j.tics.2014.04.012 24835663 PMC4112145

[IMAG.a.1336-b14] Chang, C., & Glover, G. H. (2010). Time-frequency dynamics of resting-state brain connectivity measured with fMRI. NeuroImage, 50(1), 81–98. 10.1016/j.neuroimage.2009.12.011 20006716 PMC2827259

[IMAG.a.1336-b15] Colangeli, S., Boccia, M., Verde, P., Guariglia, P., Bianchini, F., & Piccardi, L. (2016). Cognitive reserve in healthy aging and Alzheimer’s disease: A meta-analysis of fMRI studies: A meta-analysis of fMRI studies. American Journal of Alzheimer’s Disease and Other Dementias, 31(5), 443–449. 10.1177/1533317516653826 PMC10852844 27307143

[IMAG.a.1336-b16] Comsa, I. M., Bekinschtein, T. A., & Chennu, S. (2019). Transient topographical dynamics of the electroencephalogram predict brain connectivity and behavioural responsiveness during drowsiness. Brain Topography, 32(2), 315–331. 10.1007/s10548-018-0689-9 30498872 PMC6373294

[IMAG.a.1336-b201] Cox, R. W. (1996). AFNI: Software for analysis and visualization of functional magnetic resonance neuroimages. Computers and Biomedical Research, 29(3), 162–173. 10.1006/cbmr.1996.0014 8812068

[IMAG.a.1336-b17] Czisch, M., Wehrle, R., Harsay, H. A., Wetter, T. C., Holsboer, F., Sämann, P. G., & Drummond, S. P. A. (2012). On the need of objective vigilance monitoring: Effects of sleep loss on target detection and task-negative activity using combined EEG/fMRI. Frontiers in Neurology, 3, 67. 10.3389/fneur.2012.00067 22557992 PMC3338067

[IMAG.a.1336-b18] Delorme, A., & Makeig, S. (2004). EEGLAB: An open source toolbox for analysis of single-trial EEG dynamics including independent component analysis. Journal of Neuroscience Methods, 134(1), 9–21. 10.1016/j.jneumeth.2003.10.009 15102499

[IMAG.a.1336-b19] Demertzi, A., Tagliazucchi, E., Dehaene, S., Deco, G., Barttfeld, P., Raimondo, F., Martial, C., Fernández-Espejo, D., Rohaut, B., Voss, H. U., Schiff, N. D., Owen, A. M., Laureys, S., Naccache, L., & Sitt, J. D. (2019). Human consciousness is supported by dynamic complex patterns of brain signal coordination. Science Advances, 5(2), eaat7603. 10.1126/sciadv.aat7603 30775433 PMC6365115

[IMAG.a.1336-b20] Desmurget, M., & Sirigu, A. (2009). A parietal-premotor network for movement intention and motor awareness. Trends in Cognitive Sciences, 13(10), 411–419. 10.1016/j.tics.2009.08.001 19748304

[IMAG.a.1336-b21] Duncan, J. (2010). The multiple-demand (MD) system of the primate brain: Mental programs for intelligent behaviour. Trends in Cognitive Sciences, 14(4), 172–179. 10.1016/j.tics.2010.01.004 20171926

[IMAG.a.1336-b22] Duncan, J. (2013). The structure of cognition: Attentional episodes in mind and brain. Neuron, 80(1), 35–50. 10.1016/j.neuron.2013.09.015 24094101 PMC3791406

[IMAG.a.1336-b23] Falahpour, M., Chang, C., Wong, C. W., & Liu, T. T. (2018). Template-based prediction of vigilance fluctuations in resting-state fMRI. NeuroImage, 174, 317–327. 10.1016/j.neuroimage.2018.03.012 29548849 PMC8328148

[IMAG.a.1336-b25] Fox, M. D., Snyder, A. Z., Vincent, J. L., Corbetta, M., Van Essen, D. C., & Raichle, M. E. (2005). The human brain is intrinsically organized into dynamic, anticorrelated functional networks. Proceedings of the National Academy of Sciences of the United States of America, 102(27), 9673–9678. 10.1073/pnas.0504136102 15976020 PMC1157105

[IMAG.a.1336-b26] Gent, T. C., Bassetti, C. L. A., & Adamantidis, A. R. (2018). Sleep-wake control and the thalamus. Current Opinion in Neurobiology, 52, 188–197. 10.1016/j.conb.2018.08.002 30144746

[IMAG.a.1336-b27] Glerean, E., Salmi, J., Lahnakoski, J. M., Jääskeläinen, I. P., & Sams, M. (2012). Functional magnetic resonance imaging phase synchronization as a measure of dynamic functional connectivity. Brain Connectivity, 2(2), 91–101. 10.1089/brain.2011.0068 22559794 PMC3624768

[IMAG.a.1336-b29] Gonzalez-Castillo, J., Fernandez, I. S., Handwerker, D. A., & Bandettini, P. A. (2022). Ultra-slow fMRI fluctuations in the fourth ventricle as a marker of drowsiness. NeuroImage, 259, 119424. 10.1016/j.neuroimage.2022.119424 35781079 PMC9377091

[IMAG.a.1336-b30] Goodale, S. E., Ahmed, N., Zhao, C., de Zwart, J. A., Özbay, P. S., Picchioni, D., Duyn, J., Englot, D. J., Morgan, V. L., & Chang, C. (2021). fMRI-based detection of alertness predicts behavioral response variability. eLife, 10, e62376. 10.7554/eLife.62376 33960930 PMC8104962

[IMAG.a.1336-b31] Goupil, L., & Bekinschtein, T. A. (2012). Cognitive processing during the transition to sleep. Archives Italiennes de Biologie, 150(2–3), 140–154. 10.12871/00039822014231 23165874

[IMAG.a.1336-b32] Harmony, T. (2013). The functional significance of delta oscillations in cognitive processing. Frontiers in Integrative Neuroscience, 7, 83. 10.3389/fnint.2013.00083 24367301 PMC3851789

[IMAG.a.1336-b33] Harmony, T., Fernández, T., Silva, J., Bernal, J., Díaz-Comas, L., Reyes, A., Marosi, E., Rodríguez, M., & Rodríguez, M. (1996). EEG delta activity: An indicator of attention to internal processing during performance of mental tasks. International Journal of Psychophysiology, 24(1–2), 161–171. 10.1016/s0167-8760(96)00053-0 8978441

[IMAG.a.1336-b34] Hill, S., & Tononi, G. (2005). Modeling sleep and wakefulness in the thalamocortical system. Journal of Neurophysiology, 93(3), 1671–1698. 10.1152/jn.00915.2004 15537811

[IMAG.a.1336-b35] Hindman, J., Bowren, M. D., Bruss, J., Wright, B., Geerling, J. C., & Boes, A. D. (2018). Thalamic strokes that severely impair arousal extend into the brainstem. Annals of Neurology, 84(6), 926–930. 10.1002/ana.25377 30421457 PMC6344294

[IMAG.a.1336-b36] Hutchison, R. M., Womelsdorf, T., Allen, E. A., Bandettini, P. A., Calhoun, V. D., Corbetta, M., Della Penna, S., Duyn, J. H., Glover, G. H., Gonzalez-Castillo, J., Handwerker, D. A., Keilholz, S., Kiviniemi, V., Leopold, D. A., de Pasquale, F., Sporns, O., Walter, M., & Chang, C. (2013). Dynamic functional connectivity: Promise, issues, and interpretations. NeuroImage, 80, 360–378. 10.1016/j.neuroimage.2013.05.079 23707587 PMC3807588

[IMAG.a.1336-b37] Hwang, K., Shine, J. M., Cole, M. W., & Sorenson, E. (2022). Thalamocortical contributions to cognitive task activity. eLife, 11, e81282. 10.7554/eLife.81282 36537658 PMC9799971

[IMAG.a.1336-b38] Jagannathan, S. R., Bareham, C. A., & Bekinschtein, T. A. (2022). Decreasing alertness modulates perceptual decision-making. Journal of Neuroscience, 42(3), 454–473. 10.1523/jneurosci.0182-21.2021 34815316 PMC8802921

[IMAG.a.1336-b39] Jagannathan, S. R., Ezquerro-Nassar, A., Jachs, B., Pustovaya, O. V., Bareham, C. A., & Bekinschtein, T. A. (2018). Tracking wakefulness as it fades: Micro-measures of alertness. NeuroImage, 176, 138–151. 10.1016/j.neuroimage.2018.04.046 29698731

[IMAG.a.1336-b40] Jeffreys, H. (1961). The theory of probability. Oxford University Press. 10.1086/286633

[IMAG.a.1336-b41] Jo, H. J., Saad, Z. S., Simmons, W. K., Milbury, L. A., & Cox, R. W. (2010). Mapping sources of correlation in resting state FMRI, with artifact detection and removal. NeuroImage, 52(2), 571–582. 10.1016/j.neuroimage.2010.04.246 20420926 PMC2897154

[IMAG.a.1336-b42] Jubera-Garcia, E., Gevers, W., & Van Opstal, F. (2021). Local build-up of sleep pressure could trigger mind wandering: Evidence from sleep, circadian and mind wandering research. Biochemical Pharmacology, 191, 114478. 10.1016/j.bcp.2021.114478 33609561

[IMAG.a.1336-b43] Kass, R., & Raftery, A. (1995). Bayes factors. Journal of the American Statistical Association, 90(430), 773–795. 10.1080/01621459.1995.10476572

[IMAG.a.1336-b44] Killgore, W. D. S., Vanuk, J. R., Knight, S. A., Markowski, S. M., Pisner, D., Shane, B., Fridman, A., & Alkozei, A. (2015). Daytime sleepiness is associated with altered resting thalamocortical connectivity. Neuroreport, 26(13), 779–784. 10.1097/wnr.0000000000000418 26177337

[IMAG.a.1336-b45] Klimesch, W. (1999). EEG alpha and theta oscillations reflect cognitive and memory performance: A review and analysis. Brain Research. Brain Research Reviews, 29(2-3), 169–195. 10.1016/s0165-0173(98)00056-3 10209231

[IMAG.a.1336-b47] Kouider, S., Andrillon, T., Barbosa, L. S., Goupil, L., & Bekinschtein, T. A. (2014). Inducing task-relevant responses to speech in the sleeping brain. Current Biology, 24(18), 2208–2214. 10.1016/j.cub.2014.08.016 25220055 PMC4175175

[IMAG.a.1336-b48] Krakauer, J. W., Ghazanfar, A. A., Gomez-Marin, A., MacIver, M. A., & Poeppel, D. (2017). Neuroscience needs behavior: Correcting a reductionist bias. Neuron, 93(3), 480–490. 10.1016/j.neuron.2016.12.041 28182904

[IMAG.a.1336-b49] Krawczyk, D. C. (2002). Contributions of the prefrontal cortex to the neural basis of human decision making. Neuroscience and Biobehavioral Reviews, 26(6), 631–664. 10.1016/s0149-7634(02)00021-0 12479840

[IMAG.a.1336-b50] Kuznetsova, A., Brockhoff, P. B., & Christensen, R. H. B. (2017). lmerTest Package: Tests in linear mixed effects models. Journal of Statistical Software, 82, 1–26. 10.18637/jss.v082.i13

[IMAG.a.1336-b51] Larson-Prior, L. J., Zempel, J. M., Nolan, T. S., Prior, F. W., Snyder, A. Z., & Raichle, M. E. (2009). Cortical network functional connectivity in the descent to sleep. Proceedings of the National Academy of Sciences of the United States of America, 106(11), 4489–4494. 10.1073/pnas.0900924106 19255447 PMC2657465

[IMAG.a.1336-b52] Laureys, S. (2005). The neural correlate of (un)awareness: Lessons from the vegetative state. Trends in Cognitive Sciences, 9(12), 556–559. 10.1016/j.tics.2005.10.010 16271507

[IMAG.a.1336-b53] Lee, & Wagenmakers, E. J. (2014). Bayesian cognitive modeling: A practical course. Cambridge University Press. 10.1017/cbo9781139087759

[IMAG.a.1336-b54] Liao, C. H., Worsley, K. J., Poline, J.-B., Aston, J. A. D., Duncan, G. H., & Evans, A. C. (2002). Estimating the delay of the fMRI response. NeuroImage, 16(3 Pt 1), 593–606. 10.1006/nimg.2002.1096 12169246

[IMAG.a.1336-b55] Magnin, M., Rey, M., Bastuji, H., Guillemant, P., Mauguière, F., & Garcia-Larrea, L. (2010). Thalamic deactivation at sleep onset precedes that of the cerebral cortex in humans. Proceedings of the National Academy of Sciences of the United States of America, 107(8), 3829–3833. 10.1073/pnas.0909710107 20142493 PMC2840430

[IMAG.a.1336-b56] Magri, C., Schridde, U., Murayama, Y., Panzeri, S., & Logothetis, N. K. (2012). The amplitude and timing of the BOLD signal reflects the relationship between local field potential power at different frequencies. Journal of Neuroscience, 32(4), 1395–1407. 10.1523/jneurosci.3985-11.2012 22279224 PMC6796252

[IMAG.a.1336-b57] Makowski, D., Ben-Shachar, M., & Lüdecke, D. (2019). BayestestR: Describing effects and their uncertainty, existence and significance within the Bayesian framework. Journal of Open Source Software, 4(40), 1541. 10.21105/joss.01541

[IMAG.a.1336-b58] Marek, S., & Dosenbach, N. U. F. (2018). The frontoparietal network: Function, electrophysiology, and importance of individual precision mapping. Dialogues in Clinical Neuroscience, 20(2), 133–140. 10.31887/dcns.2018.20.2/smarek 30250390 PMC6136121

[IMAG.a.1336-b59] Martin, C. G., He, B. J., & Chang, C. (2021). State-related neural influences on fMRI connectivity estimation. NeuroImage, 244, 118590. 10.1016/j.neuroimage.2021.118590 34560268 PMC8815005

[IMAG.a.1336-b61] McCormick, D. A., & Bal, T. (1994). Sensory gating mechanisms of the thalamus. Current Opinion in Neurobiology, 4(4), 550–556. 10.1016/0959-4388(94)90056-6 7812144

[IMAG.a.1336-b62] Mediano, P. A. M., Ikkala, A., Kievit, R. A., Jagannathan, S. R., Varley, T. F., Stamatakis, E. A., Bekinschtein, T. A., & Bor, D. (2021). Fluctuations in neural complexity during wakefulness relate to conscious level and cognition. bioRxiv. 10.1101/2021.09.23.461002

[IMAG.a.1336-b63] Meyer, N., Harvey, A. G., Lockley, S. W., & Dijk, D.-J. (2022). Circadian rhythms and disorders of the timing of sleep. The Lancet, 400(10357), 1061–1078. 10.1016/s0140-6736(22)00877-7 36115370

[IMAG.a.1336-b64] Morey, R. D., Romeijn, J.-W., & Rouder, J. N. (2016). The philosophy of Bayes factors and the quantification of statistical evidence. Journal of Mathematical Psychology, 72, 6–18. 10.1016/j.jmp.2015.11.001

[IMAG.a.1336-b65] Mortaheb, S., Fort, L. D., Mason, N. L., Mallaroni, P., Ramaekers, J. G., & Demertzi, A. (2024). Dynamic functional hyperconnectivity after psilocybin intake is primarily associated with oceanic boundlessness. Biological Psychiatry: Cognitive Neuroscience and Neuroimaging, 9(7), 681–692. 10.1016/j.bpsc.2024.04.001 38588855

[IMAG.a.1336-b66] Mortaheb, S., Van Calster, L., Raimondo, F., Klados, M. A., Boulakis, P. A., Georgoula, K., Majerus, S., Van De Ville, D., & Demertzi, A. (2022). Mind blanking is a distinct mental state linked to a recurrent brain profile of globally positive connectivity during ongoing mentation. Proceedings of the National Academy of Sciences of the United States of America, 119(41), e2200511119. 10.1101/2021.05.10.443428 36194631 PMC9564098

[IMAG.a.1336-b67] Naghavi, H. R., & Nyberg, L. (2005). Common fronto-parietal activity in attention, memory, and consciousness: Shared demands on integration? Consciousness and Cognition, 14(2), 390–425. 10.1016/j.concog.2004.10.003 15950889

[IMAG.a.1336-b68] Niazy, R. K., Beckmann, C. F., Iannetti, G. D., Brady, J. M., & Smith, S. M. (2005). Removal of FMRI environment artifacts from EEG data using optimal basis sets. NeuroImage, 28(3), 720–737. 10.1016/j.neuroimage.2005.06.067 16150610

[IMAG.a.1336-b69] Ogilvie, R. D. (2001). The process of falling asleep. Sleep Medicine Reviews, 5(3), 247–270. 10.1053/smrv.2001.0145 12530990

[IMAG.a.1336-b70] Pascovich, C., Castro-Zaballa, S., Mediano, P. A. M., Bor, D., Canales-Johnson, A., Torterolo, P., & Bekinschtein, T. A. (2022). Ketamine and sleep modulate neural complexity dynamics in cats. The European Journal of Neuroscience, 55(6), 1584–1600. 10.1111/ejn.15646 35263482 PMC9310726

[IMAG.a.1336-b71] Perry, B. A. L., Mendez, J. C., & Mitchell, A. S. (2023). Cortico-thalamocortical interactions for learning, memory and decision-making. Journal of Physiology, 601(1), 25–35. 10.1113/jp282626 35851953 PMC10087288

[IMAG.a.1336-b72] Portoles, O., Blesa, M., van Vugt, M., Cao, M., & Borst, J. P. (2022). Thalamic bursts modulate cortical synchrony locally to switch between states of global functional connectivity in a cognitive task. PLoS Computational Biology, 18(3), e1009407. 10.1371/journal.pcbi.1009407 35263318 PMC8936493

[IMAG.a.1336-b73] Price, C. J., & Friston, K. J. (2002). Degeneracy and cognitive anatomy. Trends in Cognitive Sciences, 6(10), 416–421. 10.1016/s1364-6613(02)01976-9 12413574

[IMAG.a.1336-b74] Putman, P., Verkuil, B., Arias-Garcia, E., Pantazi, I., & van Schie, C. (2014). EEG theta/beta ratio as a potential biomarker for attentional control and resilience against deleterious effects of stress on attention. Cognitive, Affective & Behavioral Neuroscience, 14(2), 782–791. 10.3758/s13415-013-0238-7 24379166

[IMAG.a.1336-b75] Schaefer, A., Kong, R., Gordon, E. M., Laumann, T. O., Zuo, X.-N., Holmes, A. J., Eickhoff, S. B., & Yeo, B. T. T. (2018). Local-global parcellation of the human cerebral cortex from intrinsic functional connectivity MRI. Cerebral Cortex, 28(9), 3095–3114. 10.1093/cercor/bhx179 28981612 PMC6095216

[IMAG.a.1336-b76] Seli, P., Cheyne, J. A., Barton, K. R., & Smilek, D. (2012). Consistency of sustained attention across modalities: Comparing visual and auditory versions of the SART. Canadian Journal of Experimental Psychology = Revue Canadienne de Psychologie Experimentale, 66(1), 44–50. 10.1037/a0025111 21910522

[IMAG.a.1336-b77] Setzer, B., Fultz, N. E., Gomez, D. E. P., Williams, S. D., Bonmassar, G., Polimeni, J. R., & Lewis, L. D. (2022). A temporal sequence of thalamic activity unfolds at transitions in behavioral arousal state. Nature Communications, 13(1), 5442. 10.1038/s41467-022-33010-8 PMC9481532 36114170

[IMAG.a.1336-b78] Sherman, S. M. (2016). Thalamus plays a central role in ongoing cortical functioning. Nature Neuroscience, 19(4), 533–541. 10.1038/nn.4269 27021938

[IMAG.a.1336-b79] Shine, J. M., Lewis, L. D., Garrett, D. D., & Hwang, K. (2023). The impact of the human thalamus on brain-wide information processing. Nature Reviews: Neuroscience, 24(7), 416–430. 10.1038/s41583-023-00701-0 37237103 PMC10970713

[IMAG.a.1336-b80] Song, C., & Tagliazucchi, E. (2020). Linking the nature and functions of sleep: Insights from multimodal imaging of the sleeping brain. Current Opinion in Physiology, 15, 29–36. 10.1016/j.cophys.2019.11.012 32715184 PMC7374576

[IMAG.a.1336-b81] Soplata, A. E., Adam, E., Brown, E. N., Purdon, P. L., McCarthy, M. M., & Kopell, N. (2023). Rapid thalamocortical network switching mediated by cortical synchronization underlies propofol-induced EEG signatures: A biophysical model. Journal of Neurophysiology, 130(1), 86–103. 10.1152/jn.00068.2022 37314079 PMC10312318

[IMAG.a.1336-b82] Spoormaker, V. I., Gleiser, P. M., & Czisch, M. (2012). Frontoparietal connectivity and hierarchical structure of the brain’s functional network during sleep. Frontiers in Neurology, 3, 80. 10.3389/fneur.2012.00080 22629253 PMC3354331

[IMAG.a.1336-b83] Spoormaker, V. I., Schröter, M. S., Gleiser, P. M., Andrade, K. C., Dresler, M., Wehrle, R., Sämann, P. G., & Czisch, M. (2010). Development of a large-scale functional brain network during human non-rapid eye movement sleep. Journal of Neuroscience, 30(34), 11379–11387. 10.1523/jneurosci.2015-10.2010 20739559 PMC6633325

[IMAG.a.1336-b84] Stokes, M. G., Kusunoki, M., Sigala, N., Nili, H., Gaffan, D., & Duncan, J. (2013). Dynamic coding for cognitive control in prefrontal cortex. Neuron, 78(2), 364–375. 10.1016/j.neuron.2013.01.039 23562541 PMC3898895

[IMAG.a.1336-b85] Tagliazucchi, E., & Laufs, H. (2014). Decoding wakefulness levels from typical fMRI resting-state data reveals reliable drifts between wakefulness and sleep. Neuron, 82(3), 695–708. 10.1016/j.neuron.2014.03.020 24811386

[IMAG.a.1336-b86] Tagliazucchi, E., & van Someren, E. J. W. (2017). The large-scale functional connectivity correlates of consciousness and arousal during the healthy and pathological human sleep cycle. NeuroImage, 160, 55–72. 10.1016/j.neuroimage.2017.06.026 28619656

[IMAG.a.1336-b87] Tanaka, H., Hayashi, M., & Hori, T. (1996). Statistical features of hypnagogic EEG measured by a new scoring system. Sleep, 19(9), 731–738. 10.1093/sleep/19.9.731 9122561

[IMAG.a.1336-b88] Toker, D., & Sommer, F. T. (2019). Information integration in large brain networks. PLoS Computational Biology, 15(2), e1006807. 10.1371/journal.pcbi.1006807 30730907 PMC6382174

[IMAG.a.1336-b89] Tzourio-Mazoyer, N., Landeau, B., Papathanassiou, D., Crivello, F., Etard, O., Delcroix, N., Mazoyer, B., & Joliot, M. (2002). Automated anatomical labeling of activations in SPM using a macroscopic anatomical parcellation of the MNI MRI single-subject brain. NeuroImage, 15(1), 273–289. 10.1006/nimg.2001.0978 11771995

[IMAG.a.1336-b90] van den Heuvel, M. P., & Hulshoff Pol, H. E. (2010). Specific somatotopic organization of functional connections of the primary motor network during resting state. Human Brain Mapping, 31(4), 631–644. 10.1002/hbm.20893 19830684 PMC6871083

[IMAG.a.1336-b91] Winter, O., Kok, A., & Martin, E. (1995). Auditory event-related potentials to deviant stimuli during drowsiness and stage sleep. Electroencephalography and Clinical Neurophysiology, 96, 398–412. 10.1016/0168-5597(95)00030-v 7555914

[IMAG.a.1336-b92] Xu, Y., Wokke, M., Noreika, V., Bareham, C., Jagannathan, S., Georgieva, S., Trentin, C., & Bekinschtein, T. (2023). Effects of alertness on perceptual detection and discrimination. bioRxiv. 10.1101/2023.03.21.533623 40712221

[IMAG.a.1336-b93] Yeo, B. T. T., Krienen, F. M., Sepulcre, J., Sabuncu, M. R., Lashkari, D., Hollinshead, M., Roffman, J. L., Smoller, J. W., Zöllei, L., Polimeni, J. R., Fischl, B., Liu, H., & Buckner, R. L. (2011). The organization of the human cerebral cortex estimated by intrinsic functional connectivity. Journal of Neurophysiology, 106(3), 1125–1165. 10.1152/jn.00338.2011 21653723 PMC3174820

[IMAG.a.1336-b94] Zarghami, T. S., Hossein-Zadeh, G.-A., & Bahrami, F. (2020). Deep temporal organization of fMRI phase synchrony modes promotes large-scale disconnection in schizophrenia. Frontiers in Neuroscience, 14, 214. 10.3389/fnins.2020.00214 32292324 PMC7118690

