# Supplementary material for: Brain network dynamics in the wake-sleep transition reorganize according to task engagement
Source: Imaging Neurosci (Camb). 2026 Sep 8;4:IMAG.a.1336. doi: 10.1162/IMAG.a.1336 (PMC13556789; doi:10.1162/IMAG.a.1336)
Supplement: Supplementary file 1 [file IMAG.a.1336_supp.pdf]

SUPPLEMENTARY MATERIAL

		Effect Size (Drowsy > Alert)		
		**Thalamocortical**	**Auditory**	**Frontoparietal**
**Specific to Active Engagement**	Active	0.084 ± 0.003	0.078 ± 0.001	0.08 ± 0.002
	Passive	0.031 ± 0.004	0.018 ± 0.003	0.015 ± 0.003
**Specific to Passive Listening**	Active	0.028 ± 0.003	0.031 ± 0.007	.013 ± 0.005
	Passive	0.076 ± 0.001	0.069 ± 0.003	0.069 ± 0.001
**Shared by both levels of task engagement**	Active	0.086 ± 0.002	0.068	0.087 ± 0.006
	Passive	0.083 ± 0.002	0.071	0.068 ± 0.002

Table S1: Cohen's d effect sizes of ROI edges displayed in Figures 2 and 3. For the ROI edges that were Specific to Active Engagement (top row), Specific to Passive Listening (middle row), and Shared by both levels of task engagement (bottom row) in the *Drowsy>Alert* contrast, we calculated the mean and standard error of the mean Cohen's d effect sizes (Feingold, 2009).

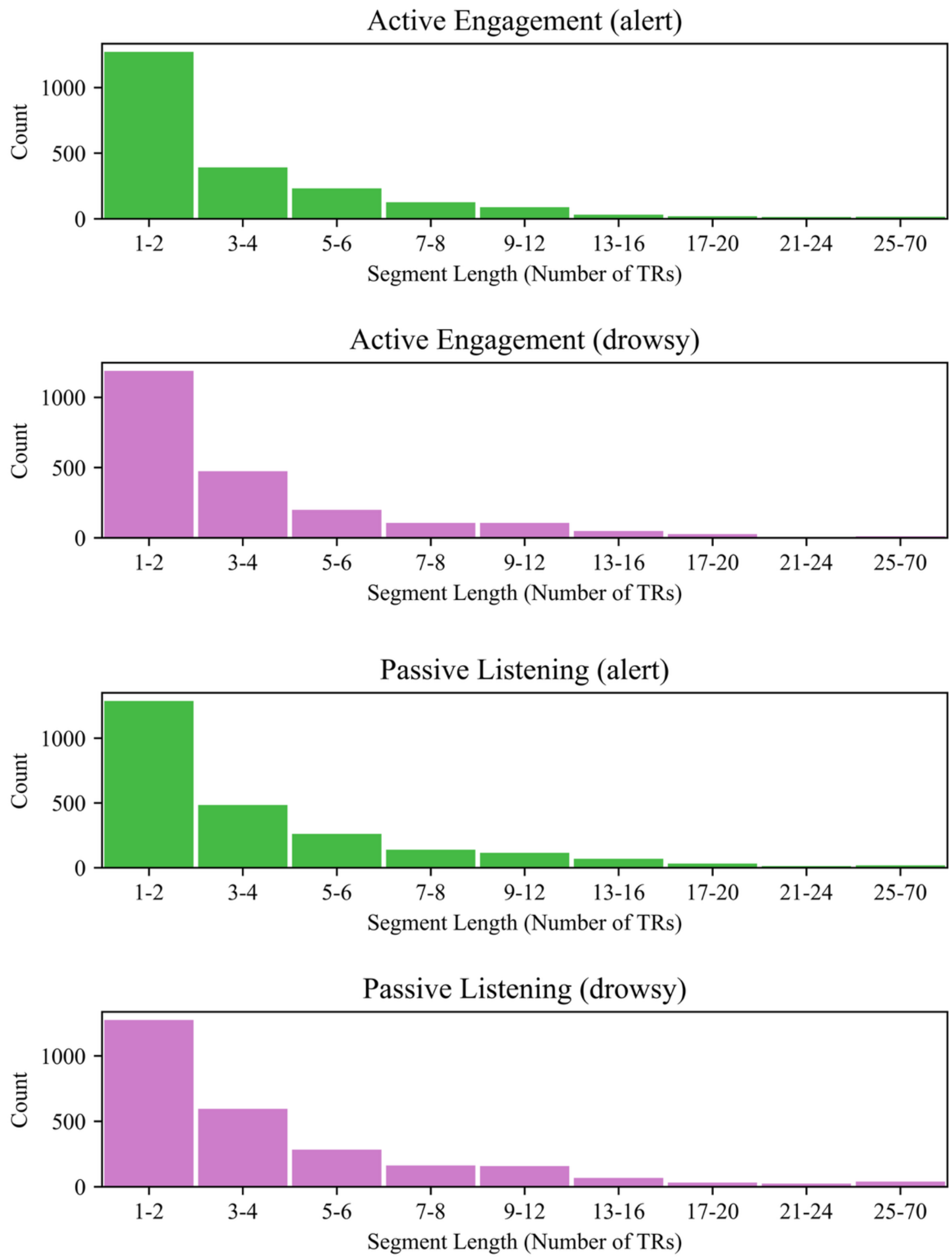

Figure S1: Distribution of same-state segment lengths after data selection: Distribution of consecutive same-state segment lengths within each dataset and state after applying data selection criteria (see **Methods 2.2.5 Data Selection Criteria**, for details).

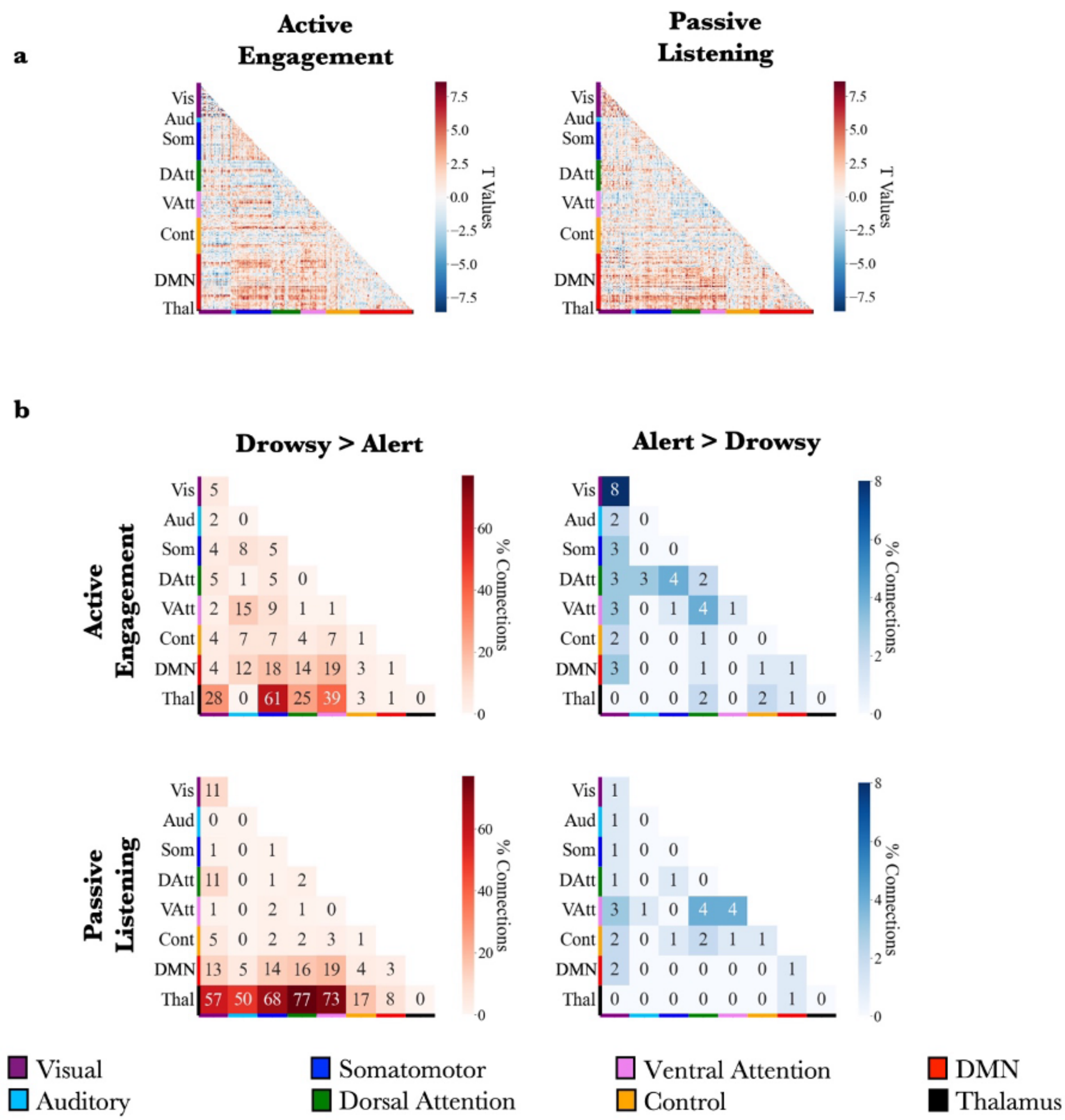

Figure S2: Alertness level predicts phase coherence. a. The effect of alertness level predicting phase coherence for each ROI edge. Higher t-value denotes *Drowsy>Alert*. Low t-value denotes *Alert>Drowsy*. **b.** Each square in each matrix shows the percentage of ROI edges between two networks that had sufficient evidence in favor of the test model's prediction (i.e., *ln(BF) > 2*).

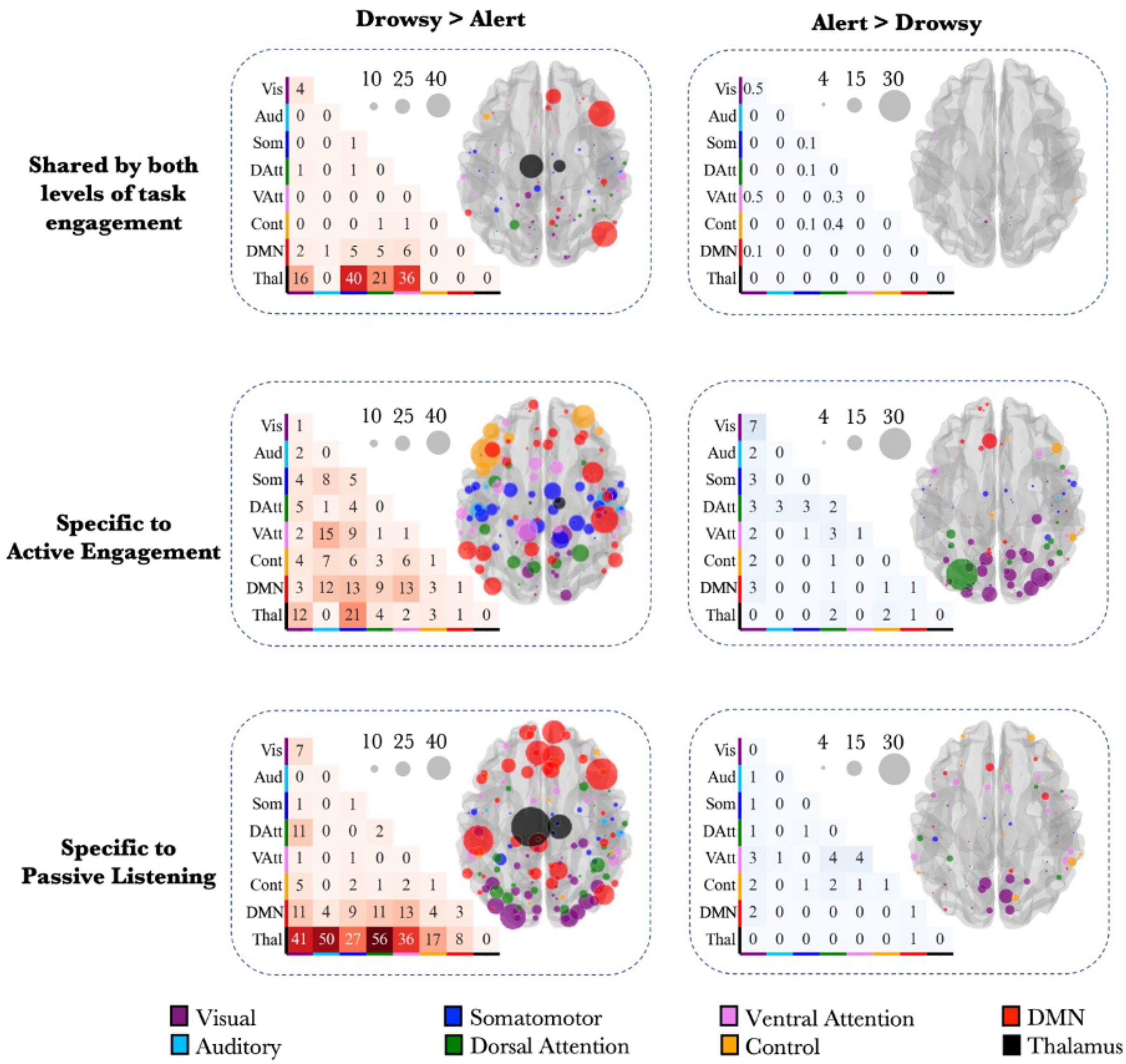

Figure S3: Whole-brain changes in phase coherence. Each square in the matrix denotes the percentage of edges between two networks that had sufficient evidence in favor of the test model's prediction. Each glass brain shows the number of edges that had sufficient evidence in favor of the test model, for each ROI node. A larger node in the glass brain denotes a higher number of edges connecting with that node. **(Top row)** Changes in phase coherence that were shared by both levels of task engagement. **(Middle row)** Phase coherence changes specific to *Active* task. **(Bottom row)** Phase coherence changes specific to *Passive* task.

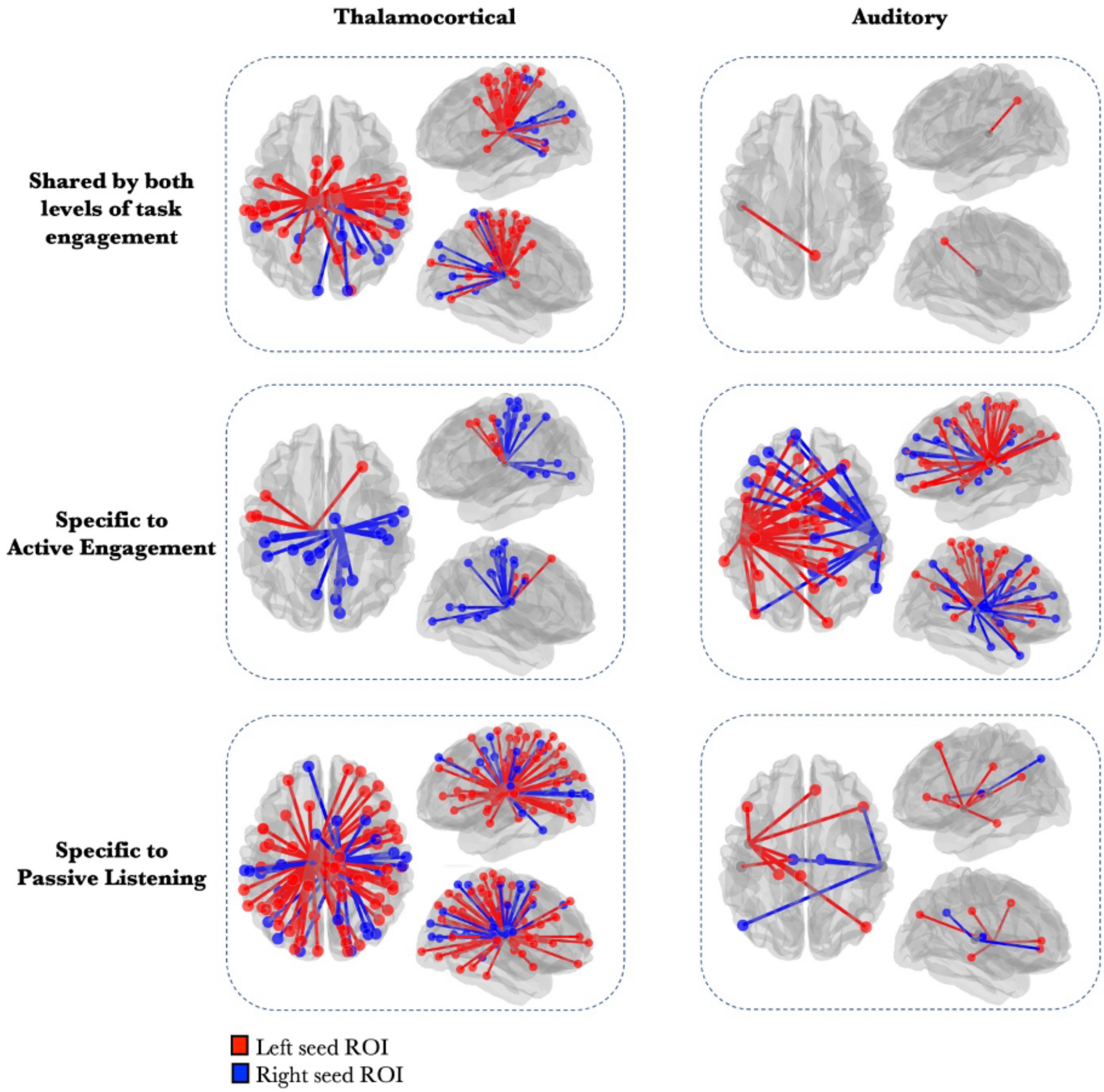

Figure S4: Laterality of thalamocortical and auditory-cortical phase coherence. (Thalamocortical column) Glass brains show the thalamocortical network edges that had sufficient evidence in favor of the test model for the *Drowsy>Alert* contrast. Edge and node color denote whether the edge stems from the right or left thalamus. **(Auditory column)** Glass brains show the network edges with the Auditory module that had sufficient evidence in favor of the test model for the *Drowsy>Alert* contrast. Edge and node color denote whether the edge stems from the right or left auditory nodes. **(Top row)** Changes in phase coherence that were shared by both levels of task engagement. **(Middle row)** Phase coherence changes specific to *Active* task. **(Bottom row)** Phase coherence changes specific to *Passive* task. Thalamocortical edges shared by both levels of task engagement: 40 edges with left node, 21 edges with right node. Thalamocortical edges specific to Active Engagement: 5 edges with left node, 21 edges with right node. Thalamocortical edges specific to Passive Listening: 66 edges with left node, 41 edges with right node. Auditory edges

shared by both levels of task engagement: 1 edge with left node, 0 edges with right node. Auditory edges specific to Active Engagement: 36 edges with left node, 20 edges with right node. Auditory edges specific to Passive Listening: 8 edges with left node, 4 edges with right node.

REFERENCES

Feingold, A. (2009). Effect sizes for growth-modeling analysis for controlled clinical trials in the same metric as for classical analysis. *Psychological Methods*, *14*(1), 43–53.
